# Supplementary material for: Chicken Meal as a Fishmeal Substitute: Effects on Growth, Antioxidants, and Digestive Enzymes in Lithobates catesbeianus
Source: Animals (Basel). 2024 Jul 29;14(15):2200. doi: 10.3390/ani14152200 (PMC11310996; doi:10.3390/ani14152200)
Supplement: Supplementary file 1 [file animals-14-02200-s001.zip › animals-3102312-supplementary.pdf]

Supplement document - transcriptional sequence of genes, cds

il-10

ATGGACAAGAAGTGTGCTGTCATCACGTGCGTTCTCCTGCTCTCTACTCCTTTTATACA
AGgGTTTGCCGTTCCTCGTGGGAAGCGTTGCCTGTGTAACAAGCTATCCAATCAGGTGA
ATGTCATGGCCCTGGCTCAAATGGATGTTTATCCAAGAAGCTCTCTGTGTGAAAACACA
GAATATATTGTCACAGTAAAAAGCACCGGTGCTACAAAATGTGTCAGTTCTGACCTGA
AAGAAATAAAAGCTCTGCTCAGTGGAAAAAATCGGTTTCTGAAACACATCCCAGTGAT
CAGACACCCCTGA

il-1β

ATGGCAGTAGTTCCTGAAATAAATGACCTCCCAATGGACAGCTACAGtgaAGAAGAATTT
TATTCAGAATGCTCCACTGAAATGAAGGTCGATACTAACCACTTTGGATGGAAAGGTTG
CATTTCTTCTCAGTCAAGCTGCAGATCTAGCATAACTCGGGACATTAGAAAGCCAAGA
AAACCTATGTCTTCCTTAAAGAAAGCAATCAATTTAGTAGTAATTATTGGAAGGGTGTC
GGGAAAGAAAAATTTTGAGTGCCTGTTCGATGACCAGGATCTGCTGAATTACATCTTAG
TGGAGGaaGACATTTCCATCAGTAATGTGGATGAGACATGTGCAGCCGCACCGAAATTC
CGCTACAGTAACACAACGGTACATTTCATTCGGGACAGCAGGCAGAAGTGTCTCGCAC
TGCAGGAATACCAGGGAAATGCCCACCTTGTGGCTCTGTTTTTACAAGGAAACAACCG
TACCAGTGAAGcaaagataaACATGGGTGCCTACATCTCTTCACCTTTCAATGGACAGACAA
GGCCAGTTACATTGAGTATTGTAGGTCGCAACCTCTATTTATCTTGCGGAGTTGAAGAG
GGAGACCAAGATACTCCAGTGTTATCACTAACGGAGGTTGAAAACATCCAGGAAAAG
AAGAATAATGACTTGCACCCCTTTCTGTTTTACAAAAGAATAAATGGCAATTCCAGCAA
TACCTTTGAATCAGTTGCTTACCCTGGCTGGTATATTTGTACCTCGCAGGCTGAAAACC
AATTTGTGCAAATGAAACCACAAAGTGATCAAGAGCACATAAAGGATTTCCTGCTTTAT
TCACCCCAATAA

il-8

CTGGAAATTCCTCTTTCGCAACCCAGCTGGAATATAAGGGGCTAGTCTCTTGCTGTTTG
AGCAGTACTCTTCAGACACAACTGAAAACCACAGCCTCAGTCATAACCTGACAAGAA
CACTTCTCACAATGAAGGCTACACTGTGTATCCTTGCAGTTCTGGCTGTCTTCCTGACA
TGTTTTACTCTCTCAGAAGggaagCCGGTTGTATCAGCACAAGAGCTCAGGTGCCAATGT
ATAAAAACAGAGAGCAAGCAAATATCTGGCAAACATTTCCTGAATCTCGAGGTGATTC
CAAAAGGCCCACACTGCAAGCATGTGGAGGTCATTGCCACCATGAAAAGTGGTCTGC
AAGTGTGTTTAGAGCCTTCTGCTCCATGGGTCAAAAAGATCATCGACAGATTTCTTGAT
ACAGCCAAAACCACTTCATCTTAATTATCACCGGACTTTACTTGACTAGTAAAAAAAGA
GAAGTTTCAAAAGAAGGCTTACTTCAGTTCACATTTACAGAAGACACATCTCCTGACC
TTAATGAATCCATATATGTGTCCATCCTGGAAATATGGACCCTAAGGGCATTGCCAGAGA
CTGAAATATACAGCCTGAAGCAAATATGTCAACTGCTGTAATCAGTGTTTTCTTTTTCTT
GCCTTATCAGATATGTATTATTACAGAGCCATGTATAGGTTTGAAGAATGGAACTGACTT
TGAAGTGGAGTTAATTAACTGACTTTTTGTAGCTTGTAAGCCACCAAAATGAAAGGAA

AGTGTCTGACtgcaacaagaatgaaaaatagcACTCCAAAATATTTATTATTGCTGTTGACCCCGAG
ATCAATGGGCTGAGAATGTCTGCTGCTGGCCAGTGACATGAGATTGTTAAGCAGTTCG
ACCAAAGTCAGCCAGATTAGGTGACAATCATCTCACTTCTATGGCTAATACAGATCATG
TGTTAACAGACTGTTAAAAGCAATGCCTTAAGCCAATGATTGGGGATCAGACTAGTGTT
GTACACGTTAGATCACAAAGCCAAATTTGTGATAATTTGTATCATGAGGATCAGTGTTTA
TGGCATTTTAAGTGGAAGTCTGTTGTACAGTATTTCATGTAGCCTACCAGTATTGAAGG
GAATTATGTAACTGAAGGTAGTTACTATTTATACTGAGCAAATCGTTTTCTTTTTACTGA
CAGAAACGTAATGTATTTTGAAAAAACACTGTATTTTTGTTTTATAATGTcaagcaagttaaaaaa
aaacatttttttacactgtttgctATGTTGTGCGTACTATTTTATAGAAATAAgagtatttaaaaaatAat

il-17

agAATTCAGTTGTGTGATTTCTGCCTCTTTCTGATTCTTTCATCTGTCACAGCCAGCATC
GTGGCACTACAAGGGGTGGAGATCACCGTAGTAGAACGCTCCACCCAAATGACTCGCT
GCGTCCAATTCCAGTTCAATAACGCATTCGACTCACAGCACAATCCAGATGGGCAGCC
GTGGCAGTTTTTCTACAACAACTTTGAAGTAATTCCAGGCAATACTTACATTGTGACCG
TGCAGCACCTACCCAGACAAGAAAGACATAATGCCAAGGAGCAGGAATTCACTGTGC
CATggTGTGATAAAGGTGACATGATGCAGACAGACACATGCTGTGATCTAGggttttgTTGG
TTTCCAAATATTACTATAAACCATCAAGAGGAAAACCTAACTGTCGAATTCAGCTCACG
ATGGAACGCCAAGAAATATGGAGTACATGTAAAAGTCAGAGATTCATTAGAGATGGGA
TATGGGAATGTCATTCTCCAAGAGGGCCCACCTATTCAACGCGTACAAATTACTTTCCC
TCATATTGGAGGTTACATAGATAAGGAATGCGTGTATAACATTTCGGTGTGGCCTTACAT
GCCATCCTGCGATAATGACTGTGTTCGAGTGTACTACGTGCCTCCATGTGCTACGGAAA
TGCCACCCCCTCCAGAACCGGTAGAACGTTGGTACTTGTGGTCTGTTGCCGTCCTGCT
GACCATTCTATTGTTTGCCGGTATTTTCTTGGGGTTACACGCTAAAGATTGCTTCAACCC
TCCAGAGAAAGTCAAACCAAATCTACCAGatattcccattcctcctccaaccaaAAAGAAGGTGTGG
TTGGTGTACTCTGCAGATCATAGATACTACTTTAAAGTGGTGATCAGGCTGGCCGATTTT
ATGCGATCAGCGTGGGGCTTGGATGTGGTACTAGACCGCCTTCATGTTCTTGAAATGAG
CACAATTACTCCCATGGCTTGGTTGGACCGCCAAAAAGACGACATAGAGAAAACAAAT
GGGACAATTCTAATCCTTTGTTCCAGAGGCGTGCAAGAGAAGTGGAAGGCAATGCAG
AATGTTAAGGAGCAAAAGATGACTTTGAAGGAGGACCGAGAACATCCAATAGAGGAC
CTCTTCACCCCTGCACTATCACTTATTCTCCCAGACTTCCAGAAAGCAATGCCCTATGAT
CGCTATGTAGTAGCATACTTCAGCAGCATCTCCACTCCAGCAGACATACCCTCAGTGTT
GGAAATTTGCCCAAAATATGCATTGACTGAAAACCTGCAAGGACTTTTCTTCCGGATCC
AGAGACAAGAGCAACACCAACCACATGTACAGCTAACTGTCAGCCATGAGGAACATA
CAAGCTACCGACGTTTAGTAAAAGCGATGGAGAGGTGCAGAGAATGGCAGGAATGTA
ACTTGCGTTGGTTTGAGAAAGAGTGTTTGTTGGTACCAGTTGAGGTCACAGAAGacacag
aggaggaagaggatatggATGATAACGTCACCCGCAAAGTCTACCCATTGATTCGCTGCCCTGAA
ACCTCAGTCTCTATGTTGGAACCACTTCTTTCGATGCCAGATCTAGTAAAGGTTTTAGAT
CCTACAATTGTGATTGGAGCTTCAAGCACTCAAGTTGAACCTTGCCTGTCTAATATAGA
TCCGTTAGTGTCAGTTGTACAACCATTCGTTCAAGAAGTAACATCAGCAGTAGTCTGCA
TTCAAGAACCACGCTTGATAGGAGAAGCTCCATCGCTTGCAGAACTCAGTAAAGGTCC
ACTGAGACAAGATGTTTCTATCCAATCGGACCAAGGTGTCCTGTCTATAGAACAGCTCA
GGGAAGCCCAAGAAAGGTTTTTCCAGAATTCCTTGGAGGATCTAAATTTGTGCTCTGC
CATGAAATTTATGGATGCTCAAGGTCAAGATTTGGTGAACCCCATTTATGATATGGCTAG

AGATCAGGCACTTAAAGCTGGTGAGCAGTTCCATGAGATTTTCCTTGATGGTCAGTTAA
CTACCCCTGGTACAGGTCTGCCCGATAGGCATTCTGTACAACTTGCAGATCAGGGATAC
AGCACATGGAACCCATATGAGGTGGACTTGAAAGCAATACAAATGGAGTCATTAAAAC
TCTTTCTGCAAAATGAGGGGGGCTTACAGAACTTTGAACAAGTATGA

cat
GTCTCCTTCCTGTTTTCGGACCGCGGTATCCCAGATGGCCACAGGCACATGAATGGTTA
CGGCTCACACACTTTCAAATTGGTTAATGCCAAGGGTGAACCTATCTACTGCAAGTTCC
ACTTCAAGcagTCCAGTGAGTNGGGCATCAGGAATCTGACGGTGGAGGAAGCCAATCG
ATTGTCTGCTGAAGATCCTGATTACGGAATCCACGATCTGTATGAAGCCATTGCCAATG
GGAACTATCCTTCCTGGTCCTTCTACATCCAGGTCATGACCTTCGAGCAAGCAGAGAG
ATACCCATTCAATCCTTTTGACTTAACTAAGATCTGGCCACACAAGGACTATCCTCTCAT
CCCTGTGGGAAAGTTGGTCCTGAACCGCAACCCGACCAACTACTTTGCAGAAGTGGA
GCAAATTGCCTTTGATCCAAGCAACATGCCTCCCGGAATCGAGCCGAGCCCGGATAAA
ATGCTGCAGGTTGACCCCTTAGTAGATGATCAGAGCAGGTCCCAGGCTGCTAAACGCT
GA

sod
GATGTTACTGCTCAGATTTCCCTCCAGCCTGCTCTTAAATTTAATGGTGGAGGCCATGTA
AATCACTCAATTTTCTGGACAAACCTCTCTCCCAGCGGTGGAGGAGAACCCCAAGGTG
AACTTTTGAAAGCCATAACAAGCGACTTTGGTTCTTTCGAGAAGTTTAAAGATAAACTT
ACAGCCATGTCAGTGGCAGTTCAGGGATCCGGATGGGGCTGGCTTGGTTATAACAAAG
AATCTAACCGCTTACAAGTCACCGCCTGTGCTAACCAAGATCCTCTGCAAGGAACAAC
AGGTCTTATTCCTCTTCTGGGCATTGATGTATGGGAGCATGCTTACTACCTGCAGTATAA
AAATGTTAGACCAGACTACCTGAAAGCCATCTGGAATGTTATCAACTGGCAGAATtttcctt
ccaacccagagcaatggcaggacgttgcagcccagtttcttgatcagtgggattttcccaactgtggtggtgctattcatggcaagcatgtccg
gattgtaccaccacccaactctggctcctattattttaattataaaggcttttgcagcatagtaaTGCTAGCTttagtgtctgccaactac
gagttctggtatctggatgttgggaagaacggccgcaactctgacTGA

gst
ATGTCTGGAAAACCCAAACTTTATTACTTTGATGGCAGAGGTAGAATGGAATCCATCCG
TTGGCTGCTTGCAGCAGCTGGAATTGAGTTTGAAGAAGAAATGTTAGAAACAAGGGA
GCAGTATGAAGCCTTACTCAAAGAAGGAGCATTACTTTTTGAGCAAGTCCCAATGGTG
CAAATGGATGGAATGAGACTTGTACAATCAAGAGCCATAATGCAGTATATGGCTGCAAA
ATATGACCTGTATGGGAAAAACATGAAGGAAAGATTgctgtatgacttttttttttacctag

sult
ATGACTATACCCAACTGGGAGCGGGCCCCTTGGCTGGAACACACGTACTTCAAGGATA
CCCTGAAGGAAGGAGAGGGCCCCAGAATCATCACCACACATCTCCCTAGTAATGTCCT
AGCACCGGCACTGCAGAACACCAAAGCTAAAGTGATCTACCTTGCCAGGAATCCGAA
GGATGTGGCGGTTTCCTTCTACTATTTCCACAAAATGGCCAAATTCCTCCCAGATTTTAA
CACCTTCTCTGAATTCCTGGACCGGTTCCTGGAGGGTAAAGTGCATTACGGATCTTGGT
TTGATCATGTGAAGGGCTGGTACCAGCAAAGAAACAACCTGGACTTCCTCTACATCAC
TTATGAAGACTTAAAGAAGGATTTGAGAAGAAGTATAAAGAAGGTGTGCCGGTTTTTG

GGCTGCCCCATGTACTCCAAAGAGGTGGATAAGGTGGAGCACCATTGCCGGTTCCCTG
AGATGAGCCAGAACATGATGGTGAATTACATGCTCATCCCCAACGAGATCCTCGACCA
CGAACAGAGCAAATTCATGAGGAAGGGCATTGTGGGTGATTGGAGGGAACACATGAC
GCCGGAGCAAAGTGACGCCTTTGATAAGATCTTCCAGGAGAAGATGTTCGGCTGCGAT
CTACAATTTCTCTGGAATTTGGACTAA
